# Evaluation of different cut-off points for IgG avidity and IgM in the diagnosis of acute toxoplasmosis in pregnant women participating in a congenital toxoplasmosis screening program

**DOI:** 10.1590/S1678-9946202466043

**Published:** 2024-07-29

**Authors:** Michelle Costa Laguardia, Ericka Viana Machado Carellos, Glaucia Manzan Queiroz Andrade, Mariângela Carneiro, José Nélio Januário, Ricardo Wagner de Almeida Vitor

**Affiliations:** 1 Secretaria de Estado de Saúde de Minas Gerais, Belo Horizonte, Minas Gerais, Brazil; 2 Universidade Federal de Minas Gerais, Instituto de Ciências Biológicas, Departamento de Parasitologia, Belo Horizonte, Minas Gerais, Brazil; 3 Universidade Federal de Minas Gerais, Faculdade de Medicina, Departamento de Pediatria, Belo Horizonte, Minas Gerais, Brazil; 4 Universidade Federal de Minas Gerais, Faculdade de Medicina, Núcleo de Ações e Pesquisa em Apoio Diagnóstico, Belo Horizonte, Minas Gerais, Brazil

**Keywords:** Toxoplasma gondii, Gestational toxoplasmosis, Diagnosis, IgG avidity, IgM

## Abstract

The main social impact of toxoplasmosis stems from its ability to be vertically transmitted. Postnatally acquired infection is generally asymptomatic in approximately 70–90% of cases, making diagnosis often dependent on laboratory tests using serological methods to search for anti-*T. gondii* antibodies. This study aimed to investigate the ability of the VIDAS TOXO IgG avidity and VIDAS TOXO IgM assays to confirm recent toxoplasmosis. In total, 341 pregnant women with suspected acute toxoplasmosis were systematically monitored in the Program for Control of Congenital Toxoplasmosis in Minas Gerais State, Brazil. We conducted an observational analytical-descriptive cross-sectional study and grouped according to clinical and laboratory criteria as having acute or chronic toxoplasmosis. The VIDAS TOXO IgG avidity and VIDAS TOXO IgM assays were evaluated to investigate the capacity to identify acute infection. IgG avidity showed good performance in identifying acute toxoplasmosis when the IgG avidity index was lower than or equal to 0.1. Values greater than or equal to 3.16 according to the TOXO IgM kit were associated with a greater chance of acute infection. These results may contribute to a more adequate diagnosis of acute gestational toxoplasmosis and, consequently, the avoidance of inadequate or unnecessary treatments.

## INTRODUCTION

*Toxoplasma gondii* is widely distributed worldwide. Approximately 25 to 30% of the world’s population is infected with the parasite^1^. *T. gondii* infection is common in pregnant women in South America, with prevalence rates from 50 to 80%^2^. In pregnant women in Brazil, the seroprevalence has been determined to range from 36% to 92%^3^. The main social impact of toxoplasmosis is associated with vertical transmission^4^. Congenital toxoplasmosis can lead to a wide variety of manifestations in fetuses and infants, including miscarriage, stillbirth, and encephalomyelitis^5^. In Brazil, specifically in Minas Gerais State, congenital toxoplasmosis seems to be a neglected disease, given its high prevalence and its concentration in municipalities with the worst socioeconomic indices^6^. Postnatally acquired infection is generally asymptomatic in approximately 70–90% of cases, making the diagnosis often dependent on laboratory tests using serological methods to search for anti-*T. gondii* antibodies^7^. However, such tests have limitations in establishing an adequate diagnosis of acute toxoplasmosis^8^. IgG avidity, a method based on the progressive increase in the affinity of the antibody to its specific antigen during the natural evolution of the immune response after infection, was determined by an auxiliary procedure to confirm or exclude a recent *T. gondii* infection. Hedman *et al*.^9^ were the first to propose the IgG-avidity test to help diagnose acute toxoplasmosis, followed by Camargo *et al.* ^10^, who applied this test to a larger sample. To improve the diagnostic capacity of the Liaison XL IgG avidity assay (DiaSorin, Saluggia, Italy), new cut-off points for anti-*T. gondii* IgG avidity were evaluated for it to be used as a marker of acute toxoplasmosis in pregnant women^11^. Very low avidity indices (<0.1) are highly suggestive of acute toxoplasmosis and can be used for treatment recommendations in pregnant women.

The Program for Control of Congenital Toxoplasmosis (PCTC-MG), initiated in the Minas Gerais State, Brazil, in 2013, included prenatal screening for toxoplasmosis and neonatal screening of children of susceptible mothers. From 2013 to 2017, 1,147 pregnant women who participated in the program were tested by the VIDAS TOXO IgG-avidity and VIDAS TOXO IgM assays (bioMérieux SA, Lyon, France). Based on the manufacturer’s instructions, 341 pregnant women were suspected of having acute toxoplasmosis and were systematically monitored by the PCTC-MG. This study aims to investigate the ability of the VIDAS TOXO IgG avidity and VIDAS TOXO IgM assays for recent infection confirmation when IgG avidity index values are very low or when IgM index values are very high.

## MATERIALS AND METHODS

This was an observational, analytical, cross-sectional study initially involving 1,147 pregnant women with suspected acute toxoplasmosis presenting positive (*i* ≥ 0.65) or inconclusive (0.55≤ *i* <0.65) anti-*T. gondii* IgM by the VIDAS TOXO IgM assay. Of the total, 341 of this pregnant women were systematically monitored during pregnancy at the PCTC-MG from 2013 to 2017 and were included in this study. Due to the absence of complete monitoring, 806 pregnant women were excluded from the study. This study evaluated the accuracy of serological tests used for prenatal screening (VIDAS TOXO IgG avidity and VIDAS TOXO IgM) to indicate acute *T. gondii* infection. A strategy similar to that of Boquel *et al.* ^11^ was used to accomplish the objective, evaluating different cut-off points for the IgG avidity index. We also investigated different cut-off points for IgM. In both cases, (IgG avidity and IgM), the accuracy of the test in evaluating the acute phase of toxoplasmosis was determined. The study protocol was reviewed and approved by the local Ethics Committee and registered on the Plataforma Brasil for ethics in research with human beings (CAAE 17907419.0.0000.5149).

### Determination of IgG and IgM levels

To determine the levels of anti-*T. gondii* IgG and IgM, the assays by VIDAS TOXO (bioMérieux SA, Lyon, France) were used in an automated system in which immunoglobulin measurement is combined with immunoenzymatic method by immunocapture with final fluorescence detection (ELFA). Based on the manufacturer’s instructions, the IgG results were expressed as units per milliliter (IU/mL) and interpreted as negative when the IU was < 4; intermediate/equivocal when 4 ≤ IU < 8; and positive when the IU was ≥8. The IgM results are expressed as IgM index. According to manufacturer of the VIDAS TOXO IgM kit, IgM index of a sample is a calculated test value, which consists of the relation between the Relative Fluorescence Value (RFV) of this sample and the memorized calibrator value, based on human control samples. IgM index *(i)* were interpreted as negative*: i* < 0.55, positive: *i* ≥ 0.65, and inconclusive: *i* between 0.55 and 0.65.

### Anti-*T. gondii* IgG avidity measurements

The VIDAS TOXO IgG avidity assay was used according to the manufacturer’s instructions. VIDAS TOXO IgG Avidity is a technique which enables low avidity antibodies to be differentiated from high avidity antibodies. The addition of urea—which disrupts the antigen-antibody (Ag-Ab) link—was found to have little effect on the high avidity Ag-Ab link, but great effect on that of low avidity antibody. Comparison of results obtained with and without a dissociating agent corresponds to one measure of avidity. The ratio between the quantity of high avidity antibodies and the quantity of total antibodies provides the index indicating antibody avidity in the tested sample (avidity index). To evaluate different cut-off points, the IgG avidity index *(ai*) was stratified as follows: *ai* ≤ 0.1 – very low avidity; 0.1 ≥ *ai* ≤ 0.2 – low avidity; 0.2 ≥ *ai* ≤ 0.3 – intermediate avidity; and *ai* > 0.3 – high avidity. Additionally, based on the manufacturer’s instructions, *ai* ≥ 0.3 exclude *T. gondii* infection acquired in the last four months.

### Definition of the serological profile of pregnant women

The gold standard for the diagnosis of acute or chronic toxoplasmosis in pregnant women was based on laboratory criteria according to Boquel *et al*.^11^. At least one of the following criteria was considered to diagnose acute toxoplasmosis: a) pregnant woman with seroconversion of anti*-T. gondii* antibody in two successive blood collections or b) pregnant woman with a significant increase in specific IgG based on an ascending curve, i.e., an increase in IgG levels of at least two times. In addition to these indicators, confirmation of vertical transmission of *T. gondii* to the newborn was also used as an indicator of acute toxoplasmosis. To confirm the occurrence of congenital toxoplasmosis in the newborn, positive results in the IgA or IgM tests were considered specific for *T. gondii* in the serum of the newborn or for the presence of IgG associated with symptoms suggestive of congenital toxoplasmosis in the newborn in addition to persistent positivity for IgG antibodies at the end of 12 months of the infant’s life.

To determine whether the pregnant woman had chronic toxoplasmosis, the stability of IgG antibodies was analyzed in two successive blood collections, based on the kinetics of antibody production, in which stable rates of IgG observed at three-week intervals are necessary to determine the stability of the production of this antibody^11^.

### Statistical analysis

The OpenEpi Program (version 3.01) was used for the following statistical analyses: positive likelihood ratio (LR+), positive predictive value (PPV), sensitivity, specificity, and kappa index. The remaining statistical analyses were performed using GraphPad Prism 5.00 Program (Prism Software, Irvine, CA, USA). The observed differences were considered statistically significant when p value < 0.05.

## RESULTS

All 341 pregnant women with suspected acute toxoplasmosis were included in the analysis of the IgM accuracy test for identifying acute maternal infection, and 339 were included in the evaluation of the IgG avidity test. Acute toxoplasmosis profile was observed in 120/146 (82.2%) pregnant women with an *ai* ≤ 0.1; in 52/79 (65.8%) pregnant women with an *ai* between 0.1 and 0.2; in 10/39 (25.6%) pregnant women with an *ai* between 0.2 and 0.3; and in 17/75 (22.7%) pregnant women with an *ai* ≥ 0.3 (p < 0.0001; χ^2^ = 92.64, 3gl) (Table 1).

**Table 1:** Estimation of acute or chronic toxoplasmosis according to the IgG avidity index ranges: very low (≤ 0.1), low (0.1 – 0.2), intermediate (0.2 – 0.3) and high (≥ 0.3) by the VIDAS TOXO IgG-avidity assay from 2014 to 2017.

IgG avidity (*ai*)	Acute toxoplamosis* (%)	Chronic toxoplasmosis** (%)	Total
≤ 0.1	120 (82.2%)	26 (17.8%)	146
0.1–0.2	52 (65.8%)	27 (34.2%)	79
0.2–0.3	10 (25.6%)	29 (74.4%)	39
≥ 0.3	17 (22.7%)***	58 (77.3%)	75
Total	199	140	339

*Defining criteria for acute toxoplasmosis: a) Seroconversion of anti*-T. gondii* IgG antibody in two successive blood collections, and/or b) significant increase in specific anti-*T. gondii* IgG based on an ascending curve; and/or c) confirmation of the occurrence of congenital toxoplasmosis in the newborn (see Materials and Methods); **Anti-*T. gondii* IgG stability in two successive blood collections; ***Eight pregnant women presented IgM seroconversion with high IgG avidity in the second blood collection within a 2-month interval.

The highest sensitivity (60.3%) and specificity (81.4%) values for identifying acute *T. gondii* infection were obtained at the cut-off point *ai* ≤ 0.1 (Table 2). Additionally, at this cut-off point, the highest values of LR+ (3.24), PPV (82.2%), and moderate concordance (0.4) were observed.

**Table 2:** Accuracy analysis of IgG avidity index cut-off points for identifying acute *T. gondii* infection in pregnant women who underwent serology at PCTC-MG using the VIDAS TOXO IgG-avidity assay from 2014 to 2017.

IgG avidity index (*ai*)	Sensitivity% (95% CI)	Specificity% (95% CI)	LR+ (95% CI)	PPV % (95% CI)	Kappa (95% CI)
≤ 0.1	60.3 (53.4 – 66.8)	81.4 (74.2 – 87.0)	3.2 (3.0 – 3.5)	82.2 (75.2 – 87.5)	0.4 (0.3 – 0.5)
[0.1– 0.2]	26.1 (20.5 – 32.6)	80.7 (73.3 – 86.4)	1.4 (1.1 – 1.6)	65.8 (54.8 – 75.3)	0.06 (−0.02 – 0.1)
[0.2 – 0.3]	5.0 (2.7 – 9.0)	79.3 (71.8 – 85.2)	0.2 (0 – 10.5)	25.6 (14.6 – 41.1)	− 0.1 (−0.2 – −0.01)
≥ 0.3	8.5 (5.4 – 13.3)	58.6 (50.3 – 66.4)	0.2 (0.1 – 0.7)	22.7 (14.7 – 33.4)	− 0.3 (−0.4 – −0.2)

LR+ = Likelihood ratio; PPV = Positive Predictive Value.

Of the 163 pregnant women with an IgM index (*i*) ≥ 3.16 (upper quartile), 128 (78.5%) had an acute toxoplasmosis profile (p < 0.0001 χ^2^ = 49.49, 1gl) (Table 3). On the other hand, only 73 (41%) of the 178 pregnant women with an *i* between 0.65 and 3.15 had acute toxoplasmosis. Compared with the other quartiles, the IgM quartile that included *i* ≥ 3.16 presented the highest sensitivity and specificity values, of 63.7% and 75.0%, respectively (Table 4). Additionally, the highest values of LR+ (2.5), PPV (78.5), and poor concordance (0.37) were observed at this cut-off point.

**Table 3:** Estimation of acute or chronic toxoplasmosis according to the intervals of the IgM index (*i*) by the VIDAS TOXO IgM assay from 2014 to 2017.

IgM index (*i*) quartiles	Acute toxoplasmosis* (%)	Chronic toxoplamosis** (%)	Total
0.65–1.01	13 (30.2%)	30 (69.8%)	43
1.02–1.64	17 (29.8%)	40 (70.2%)	57
1.65–3.15	43 (55.1%)	35 (44.9%)	78
3.16–18.40	128 (78.5%)	35(21.5%)	163
Total	201	140	341

*Defining criteria for acute toxoplasmosis: a) Seroconversion of anti*-T. gondii* IgG antibody in two successive blood collections, and/or b) significant increase in specific anti-*T. gondii* IgG based on an ascending curve; and/or c) confirmation of the occurrence of congenital toxoplasmosis in the newborn (see Materials and Methods); **Anti-*T. gondii* IgG stability in two successive blood collection.

**Table 4:** Accuracy analysis of IgM cut-off points for identifying acute *T. gondii* infection in pregnant women who underwent serology at PCTC-MG using the VIDAS TOXO IgM assay from 2014 to 2017.

IgM index (i) quartiles	Sensitivity% (95% CI)	Specificity% (95% CI)	LR+ (95% CI)	PPV % (95% CI)	Kappa (95% CI)
[0.65 – 1.01]	6.5 (3.8 – 10.6)	78.6 (71.1 – 84.6)	0.3 (0.3 – 2.8)	30.2 (18.6 – 45.1)	−0.13 (−0.2 – −0.07)
[1.02 – 1.64]	8.5 (5.3 – 13.1)	71.4 (63.5 – 78.3)	0.3 (0.1 – 1.1)	29.8 (19.5 – 42.7)	−0.17 (−0.2 – −0.1)
[1.65 – 3.15]	21.4 (16.3 – 27.6)	75.0 (67.2 – 81.4)	0.9 (0.7 – 1.1)	55.1 (44.1 – 65.7)	− 0.03 (−0.1 – 0.05)
[3.16 – 18.40]	63.7 (56.8 – 70.0)	75.0 (67.2 – 81.4)	2.5 (2.4 – 2.7)	78.5 (71.6 – 84.1)	0.37 (0.3 – 0.5)

LR+ = Likelihood ratio; PPV = Positive Predictive Value.

**Figure 1:**
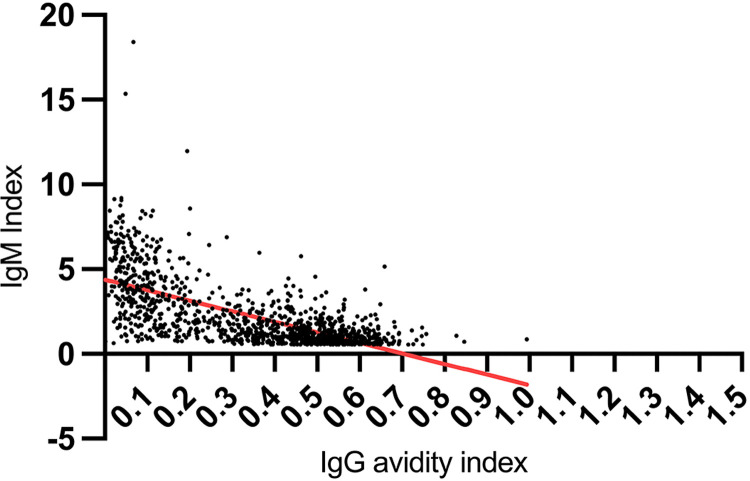
Correlation of IgG avidity index and IgM index in pregnant women with suspected acute toxoplasmosis evaluated respectively by the VIDAS TOXO IgG avidity and VIDAS TOXO IgM assays, within the PCTC-MG Program, Brazil, 2014 to 2017. R = -0.65; 95% CI −0.6868 to −0.6205, p < 0.0001.

Analysis of the 1,145 pregnant women, tested for both IgG avidity and IgM, showed an inverse correlation between the two variables, IgG avidity index and the IgM index (Pearson r = −0.65; 95% CI −0.69 to −0.62; R-squared 0.43; p < 0.0001), as shown in Figure 1. The highest IgM indices were correlated with the lowest IgG avidity index values. However, among the 1,039 pregnant women presenting positive anti-*T. gondii* IgM (*i* ≥ 0.65), 585 (56.3%) had high IgG avidity (*ai* > 0.3). Among the 106 pregnant women presenting inconclusive IgM results (0.55 ≤ *i* < 0.65), 105 (99.1%) had high IgG avidity.

## DISCUSSION

A rapid and adequate diagnosis of gestational toxoplasmosis allows for timely treatment, reducing the risk of serious neurological sequelae^12^ and the risk of intracranial lesions if treatment is given within the first four weeks following maternal infection^13^. Despite technological advances in laboratory diagnosis and the availability of serological methods for testing for anti-*T. gondii* antibodies in pregnant women, there are still many limitations in clinical practice for the prediction of the date of maternal infection. The following factors hamper the prediction of the date of maternal infection: late enrollment of pregnant women to start prenatal care and screening tests, the continued presence of residual anti-*T. gondii* IgM for long periods, and the presence of low avidity IgG in some people with chronic *T. gondii* infection^14^.

Although a low avidity result is still considered noninformative for the prediction of the infection date, some authors suggest that very low avidity results are highly suggestive of recent infections^15^. Thus, some authors have already shown interest in defining new cut-off points for anti-*T. gondii* IgG avidity using different assays. Very low values obtained by the Elecsys Toxo Avidity (Roche Diagnostics) assay could help confirm a recent infection^16^. Another study using the same kit (Elecsys Toxo Avidity) showed that IgG avidity values ≥ 90% (≥ 0.9) excluded infection in less than nine months. Furthermore, avidity values ≤15% (≤0.15) excluded infection in more than three months^17^. The Liaison XLToxo IgG avidity assay (DiaSorin, Salluggia, Italy) was more accurate in confirming acute infection when the IgG avidity index was very low (≤ 0.1)^11^. Another study using the same test (Liaison XLToxo IgG avidity) showed that an IgG avidity index above 0.11 is highly suggestive of chronic toxoplasmosis, and able to exclude acute toxoplasmosis infection in the first trimester of pregnancy with very good accuracy^18^.

Our study showed that the VIDAS TOXO IgG avidity assay achieved good accuracy in identifying acute toxoplasmosis when the IgG avidity index was lower than or equal to 0.1. We observed that 82.2% of the pregnant women with an IgG avidity index ≤ 0.1 had acute infection, while 65.8% with an IgG avidity index between 0.1 and 0.2 had acute toxoplasmosis. We also observed that among pregnant women classified as having acute infection, 25.6% had an intermediate avidity (*ai* = 0.2–0.3), and 22.7% had a high avidity (*ai* ≥ 0.3). These discordant results suggest that, in some pregnant women, the avidity test was performed late, long after maternal infection.

Eight pregnant women who showed seroconversion with high IgG avidity at the second blood collection (within a 2-month interval) were identified. The mistaken identification of these eight pregnant women who seroconverted during pregnancy and who had high IgG avidity in the second blood collection can be explained by the use of a quarterly serology screening model in Brazil. Due to the difficulty in women adhering to screening programs, the period between exams can often be even longer than three months. Similar results were observed in two patients who seroconverted, already showing high IgG avidity at the time of conclusive diagnosis^19^. In another study, seroconversion was detected in a pregnant woman with a high avidity at the second blood collection^20^. According to Lefevre-Pettazzoni *et al.* ^21^, the patterns of the evolution of IgG avidity throughout time were quite diverse among distinct pregnant women. However, the trend was toward a statistically significant increase. These authors observed, on average, a 1.0167-fold increase in IgG avidity for each additional gestational week after the putative date of infection. After birth of infants congenitally infected with *T. gondii*, IgG avidity values slowly increased over time for up to two years^22^.

When using *ai* ≤ 0.1 as the avidity cut-off point for identifying acute toxoplasmosis, reasonable sensitivity values (60.3%) and specificity values (81.4%) were observed. At other cut-off points, the sensitivity was even lower, despite reasonable specificity. At this cut-off point (*ai* ≤ 0.1), the likelihood ratio was 3.24, showing that, when using an IgG avidity index ≤ 0.1, the chance of correctly discriminating a pregnant woman with acute toxoplasmosis from pregnant women with latent toxoplasmosis is 3.24 times greater. The highest positive predictive value (82.2) was also observed at this cut-off point.

The VIDAS TOXO IgM kit has been demonstrated to be a good tool for diagnosing acute toxoplasmosis. Our results showed that IgM levels ≥ 3.16 were associated with a greater chance of acute infection. Using this cut-off point, the sensitivity was 63.7%, and the specificity was 75.0%. However, in another study, it was observed that the index value of the anti*-T. gondii* IgM antibody test cannot be considered a good predictor of recent infection^23^. It is important to highlight the distinct objectives and methodologies between the two studies, which may account for the differences in findings. Our study specific focus on pregnant women with suspected acute toxoplasmosis and the use of comprehensive laboratory criteria to define acute infection underscore the relevance of our findings in a clinical setting aimed at prenatal screening. Residual anti-*T. gondii* IgM detection also interferes with the correct diagnosis of toxoplasmosis, since patients with IgM associated with high avidity of IgG are not rare. Perhaps, if our sample of pregnant women acutely infected with *T. gondii* were systematically followed up after delivery, we could identify a significant number of cases presenting residual IgM, associated with high IgG avidity, as shown by Ribeiro *et al.* ^24^, that observed pregnant women with high avidity IgG simultaneously with residual IgM.

The correlation between the variables of IgG avidity and the IgM index was significant, showing that the greater the IgM level was, the lower the IgG avidity. Similar observations have already been made showing a moderate or inverse correlation between IgM and IgG avidity in serum samples with low IgG avidity^25^. However, our study showed a significant number of discordant results between anti-*T. gondii* IgM and IgG avidity: we observed pregnant women positive for IgM with high IgG avidity. Thus, positive anti-*T. gondii* IgM must be interpreted with caution in the diagnosis of toxoplasmosis, as already described^26^. In a recent study, the combination of anti-*T. gondii* IgM and IgG avidity assays was shown to be a reliable and suitable method for diagnosing acute toxoplasmosis^27^.

Given the importance of early treatment, our results are relevant for clinical practice and can assist healthcare professionals in deciding to immediately start treatment for pregnant women evaluated by the VIDAS TOXO IgG avidity and IgM assays, showing IgG avidity ≤ 0.1 and IgM indices ≥ 3.16 values. This serological profile can also be used as an indication for enhanced ultrasound surveillance of fetal development and/or testing for *T. gondii* in amniotic fluid by PCR.

In this work, we present evidence supporting the use of VIDAS TOXO IgG avidity assay to indicate acute infection by *T. gondii* in pregnant women with very low IgG avidity indices (≤ 0.1). Thus, we suggest that in screening for toxoplasmosis in pregnant women in the PCTC-MG, an IgG avidity index ≤ 0.1 should be considered a strong indicator of acute toxoplasmosis, associated or not with an IgM index ≥ 3.16.

This study was based on the PCTC-MG database, which stores information from prenatal serological screening of pregnant women treated by the program. Data stored in this bank come from the pregnant women’s medical history and are entered manually. This methodology could explain the loss of some information related to gestational age at the time of the first blood sample collection and the lack of conclusive diagnosis of congenital toxoplasmosis in some newborns. The limitations found were mainly caused by inadequate data filling by health professionals regarding the pregnant women, and the typing errors inherent in this form of data entry.

## CONCLUSION

Our results allow us to identify a serological profile that provides greater clarity regarding the time of *T. gondii* infection and the therapeutic indications for treatment. Very low IgG avidity indices (≤ 0.1) obtained by the VIDAS TOXO IgG avidity assay, as well as very high IgM indices (≥ 3.16) obtained by the VIDAS TOXO IgM assay, are important markers in the diagnosis of acute toxoplasmosis.

## References

[1] Montoya JG, Liesenfeld O (2004). Toxoplasmosis. Lancet.

[2] Bigna JJ, Tochie JN, Tounouga DN, Bekolo AO, Ymele NS, Youda EL (2020). Global, regional, and country seroprevalence of Toxoplasma gondii in pregnant women: a systematic review, modelling and meta-analysis. Sci Rep.

[3] Dubey JP, Lago EG, Gennari SM, Su C, Jones JL (2012). Toxoplasmosis in humans and animals in Brazil: high prevalence, high burden of disease, and epidemiology. Parasitology.

[4] Remington JS, McLeod R, Thulliez P, Desmonts G, Remington JS, Klein JO, Baker CB, Wilson CJ (2006). Infectious diseases of the fetus and newborn.

[5] Dubey JP, Murata FH, Cerqueira-Cézar CK, Kwok OC, Villena I (2021). Congenital toxoplasmosis in humans: an update of worldwide rate of congenital infections. Parasitology.

[6] Carellos EV, Andrade GM, Vasconcelos-Santos DV, Januário JN, Romanelli RM, Abreu MN (2014). Adverse socioeconomic conditions and oocyst-related factors are associated with congenital toxoplasmosis in a population-based study in Minas Gerais, Brazil. PLoS One.

[7] Robert-Gangneux F, Dardé ML (2012). Epidemiology of and diagnostic strategies for toxoplasmosis. Clin Microbiol Rev.

[8] Villard O, Cimon B, L´Ollivier C, Fricker-Hidalgo H, Godineau N, Houze S. (2016). Serological diagnosis of Toxoplasma gondii infection: recommendations from the French National Reference Center for Toxoplasmosis. Diagn Microbiol Infect Dis.

[9] Hedman K, Lappalainen M, Seppäiä I, Mäkelä O (1989). Recent primary Toxoplasma infection indicated by a low avidity of specific IgG. J Infect Dis.

[10] Camargo ME, Silva SM, Leser PG, Granato CH (1991). Avidez de anticorpos IgG específicos como marcadores de infecção primária recente pelo Toxoplasma gondii. Rev Inst Med Trop Sao Paulo.

[11] Boquel F, Monpierre L, Imbert S, Touafek F, Courtin R, Piarroux R (2019). Interpretation of very low avidity indices acquired with the Liaison XL Toxo IgG avidity assay in dating toxoplasmosis infection. Eur J Clin Microbiol Infect Dis.

[12] Cortina-Borja M, Tan HK, Wallon M, Paul M, Prusa A, Buffolano W (2010). Prenatal treatment for serious neurological sequelae of congenital toxoplasmosis: an observational prospective cohort study. PLoS Med.

[13] Gras L, Wallon M, Pollak A, Cortina-Borja M, Evengard B, Hayde M (2005). Association between prenatal treatment and clinical manifestations of congenital toxoplasmosis in infancy: a cohort study in 13 European centres. Acta Paediatr.

[14] Kodym P, Kurzová Z, Berenová D, Malý M (2023). Detection of persistent low IgG avidity-an interpretative problem in the diagnosis of acute toxoplasmosis. PLoS One.

[15] Garnaud C, Fricker-Hidalgo H, Evengård B, Álvarez-Martínez MJ, Petersen E, Kortbeek LM (2020). Toxoplasma gondii-specific IgG avidity testing in pregnant women. Clin Microbiol Infect.

[16] Murat JB, L'Ollivier C, Hidalgo HF, Franck J, Pelloux H, Piarroux R (2012). Evaluation of the new Elecsys Toxo IgG avidity assay for toxoplasmosis and new insights into the interpretation of avidity results. Clin Vaccine Immunol.

[17] Fricker-Hidalgo H, L'Ollivier C, Bosson C, Imbert S, Bailly S, Dard C (2017). Interpretation of the Elecsys Toxo IgG avidity results for very low and very high index: study on 741 sera with a determined date of toxoplasmosis. Eur J Clin Microbiol Infect Dis.

[18] Skvarc M (2022). Diagnostic accuracy of adjusted low IgG avidity index to predict acute Toxoplasma gondii infection in the first trimester of pregnancy. Folia Parasitol. (Praha).

[19] Jenum PA, Stray-Pedersen B, Gundersen AG (1997). Improved diagnosis of primary Toxoplasma gondii infection in early pregnancy by determination of antitoxoplasma immunoglobulin G avidity. J Clin Microbiol.

[20] Fricker-Hidalgo H, Cimon B, Chemla C, Darde ML, Delhaes L, L'Ollivier C. (2013). Toxoplasma seroconversion with negative or transient immunoglobulin M in pregnant women: myth or reality? a French multicenter retrospective study. J Clin Microbiol.

[21] Lefevre-Pettazzoni M, Bissery A, Wallon M, Cozon G, Peyron F, Rabilloud M (2007). Impact of spiramycin treatment and gestational age on maturation of Toxoplasma gondii immunoglobulin G avidity in pregnant women. Clin Vaccine Immunol.

[22] Buffolano W, Lappalainen M, Hedman L, Ciccimarra F, Del Pezzo M, Rescaldani R (2004). Delayed maturation of IgG avidity in congenital toxoplasmosis. Eur J Clin Microbiol Infect Dis.

[23] Bakir A, Guney M (2022). Determination of low IgG class antibody avidity percentage by IgM levels specific to Toxoplasma gondii. Clin Lab.

[24] Ribeiro AC, Mutis MS, Fernandes O (2008). Association of the presence of residual anti-Toxoplasma gondii IgM in pregnant women and their respective family groups in Miracema, Northwest Rio de Janeiro, Brazil. Mem Inst Oswaldo Cruz.

[25] Reis MM, Tessaro MM, D'Azevedo PA (2006). Toxoplasma-IgM and IgG-avidity in single samples from areas with a high infection rate can determine the risk of mother-to-child transmission. Rev Inst Med Trop Sao Paulo.

[26] Liesenfeld O, Montoya JG, Kinney S, Press C, Remington JS (2001). Effect of testing for IgG avidity in the diagnosis of Toxoplasma gondii infection in pregnant women: experience in a US reference laboratory. J Infect Dis.

[27] Ikuta K, Kanno R, Bessho T, Koshizuka T, Suzutani T (2023). Evaluation of Toxoplasma gondii IgG avidity assays through a comparison of IgM serostatus. Diagn Microbiol Infect Dis.

